# Care of the Sick and Injured1Read before the Eighth District Branch of the Medical Society of the State of New York, held in Buffalo, September 8-9, 1909.

**Published:** 1909-11

**Authors:** J. C. Young

**Affiliations:** Cuba, New York


### Care of the Sick and Injured.[1]

By J. C. YOUNG, M.D., M.R.C.S., M.R.C.P., Cuba, New York.

It is said that the sick or injured deer is abandoned by the rest of the herd, that such is the instinct of those animals. But the instinct of the human race is far different. We are told there was a time when no man was his brother's keeper. While we may still see or learn in the course of a lifetime of some few instances where a heartless indifference has been manifested toward a sick or injured human being, yet it is the instinct of the human race to go out with a deep feeling of tenderness and sympathy toward such persons. History teaches us that at all times and in all ages mankind has manifested in different ways a kindly tenderness toward the sick or injured.

The natural regard felt for those who are near and dear to them in health has led to the adoption of some means or methods

1. Read before the Eighth District Branch of the Medical Society of the State of New York, held in Buffalo, September 8-9, 1909.

for their relief and safety, when unable to care for themselves. A sick or injured person welcome the flowers which bloom and fade, but how much more they welcome the care which always saves suffering and many times saves both life and health to its possessor. This help and care is the monument they desire and welcome most.

Warriors have ambitions for the luster of a single name, but this is always at the expense of a victory won or lost. But in recent years there has sprung up all over this land institutions of learning, and out from their doors have passed and are passing thousands who, through special study, have prepared themselves for a lifework which is often far more commendable than that of warriors, and that is a work of saving life and prevention of suffering.

As far back as the oldest of us remember, people were very apt to remark that it was as much in care as it was in the medicine. Long years ago members of the medical profession who practised, then worked very hard in nursing the sick back to health. In many instances great confusion if not serious trouble followed such efforts, as it was always unscientific and inaccurate, but it was the best we were able to do for our best friends. Now this is all changed for we see now that the means used with the best of intentions should be abandoned as inexact and unsafe. The physician formerly performed many of the duties which the nurse of today does. But the doctor could not remain with the case as other duties were pressing upon him, and still he could not help but realise how important it was to have the constant attention of a nurse. Vision and reality differ, for generally they are widely separated as to time and space; but the vision of the doctor, well advanced in years and still practising medicine—all the best years of his life being spent in the sick room—becomes very nearly realised now as to care of sick and injured and adds an alluring interest as they pass down the descent of life's avenues.

The great advances which have taken place in medical and surgical science has created a need for new help, both for the patient and medical attendant, a need which has been met to a great extent by the trained nurse, her coming into the sick room being an inspiration to physician, patient and friends. During recent years, a care of the sick and injured has been introduced which is far in advance of the older and so familiar methods to many of us here today. Since the change in the treatment of wounds and diseases, the whole aspect of the question has been altered.

This is certainly a decided step forward, and has a tendency to relieve the uncertainty which heretofore surrounded the case.

Some brilliant results have been obtained by prevailing methods that could not be in any other manner. It is claimed when the history of medicine of the present time comes to be written, we will be accused of endorsing almost any methods which seem to contain something new or some unusual feature. But this is not entirely new. Nursing is as old as the time when mothers first bore and cared for their offspring but the trained nurse is not taking us back very far. It is only one of the means of replacing the more unsatisfactory and unsafe methods of years ago. Nursing the sick and injured has made its mark in history to remain as very interesting pages to look back upon.

This world is indebted for a lasting and substantial benefit to many changes and improvements which have taken place in our day. The inventors claim most for their genius. The chemists also claim much. The pathologists, too, justly claim much, while other professions should not be ignored. But suffering humanity is surely indebted very much to those who, from surrounding circumstances or their own inclinations and wishes, have fitted themselves to relieve suffering and save life by the care and attention they are able to give. It means much more than good intentions which any one could give. Good intentions count for nought. They may be positively harmful. The advance in medical science and the care which a nurse now can give a sick and injured person make the great sacrifices of former times on our part needless. We are no longer expected to risk so much while we are in the pursuit of our duty.

If the nurse is so helpful in the homes where wealth furnishes every comfort money can bring, how much greater help would they be in homes where poverty is fostered in all its forms and where there is comparatively little to help the sufferer. The fact that there are a great number of sick and suffering all the time who are unable to obtain the help of a properly trained and educated nurse, has repeatedly drawn the attention of the medical profession as well as the public to this matter. The rights of the poor form a very interesting subject. The medical profession as guardians of the public health, would naturally be looked to first in regard to this, but the subject is of such great importance it is rather surprising that so far it has received so little attention from the public mind. There is a class of people who get along very well in health and yet find it difficult to make the income equal the outgo, but when sickness comes it strikes a new terror into their lives and they ask, what in this world will happen to us next? Humanity inspires us to do all within our power for the sick and injured no matter what their stations in life may be.

Things must be arranged not on personal grounds but according to the needs of the patient and nature of the disease. As human beings each case deserves the same care and consideration. Our duty and work is to restore to health or relieve the sufferings of those who have been overtaken by disease. It should never be a question of how much or how little we can do, but what ought to be done and then do our best. Lamentable as it may seem, disease sometimes causes death in a quiet way, and takes a valuable life out of the world, which might have been saved if proper care could have been given the case. While we are looking for better days to come to this class, yet present needs are crowding upon us and the question is, how shall we best provide for them now? Never before in the history of medicine has so much interest centered on this question as at the present time. The medical profession is deeply interested in it. And so we trust the time is near at hand when the needy poor may feel that by stretching out the hand, a helping hand will be found waiting to clasp their own in return. Such a fellowship constitutes a bond unbroken, by time unsealed.

People may give different opinions as to what it is to die rich, but all will agree that the man or woman died very rich who asked, when was it that I had this account placed to my credit, which has been accumulating all these years? And the answer comes, when you saw the poor woman, the little boy or girl who were sick and in destitute circumstances and you sent a nurse to care for them, until they were relieved by art or released of their sufferings by death. You had this account placed to your credit then and when you died you did not leave all your wealth in the world. As the old Scotchman said, "this world we are passing through is God's world just as much as any we are going to." How poor people can obtain the care of a nurse when sick, has within recent years received marked attention, not only at the hands of the medical profession but by some others as well. It is difficult many times, and in some cases we all must admit, but difficulties make the opposite possible. The question comes every day to both the nurse and the doctor, what can I do and what can you do to help a sick person to get well? The well trained nurse deserves and expects to receive $25.00 a week. But there are comparatively few who can afford such a price for any length of time.

But there are schools both by correspondence and schools of short training, where many can learn much as to how to help a sick person to get well and relieve suffering, but who could not afford the time or expense to take full training. Such nurses could afford to give service at less than one-half the price given by others. There should always be a full distinction made between the two classes. But I think all classes are getting nearer together on this point. I am pleased to note that with the well trained three year course nurse, a marked changed in her views and opinions has taken place on this point. All recognise more and more that the partly trained nurse is far in advance of one with no training, and can furnish that care and attention which will be a great help, at a price the full trained nurse could not afford to work for. We trust the time is near when the sick poor will have something more than desire or wish, but a reality.

The nurse is the doctor's best assistant and very much of his treatment must be carried into effect by her. In very many cases the nurse stands ahead of any or all other measures we are able to adopt, in piloting patients safely over critical periods in their lives. The doctor requests and wishes but he cannot carry out his wishes or requests. There no longer remains a doubt that the medical profession owes to nurses a debt of gratitude we can hardly repay. The most we can do is to cherish it, remember it, and acknowledge it. But we must not allow the public to feel that we wish the trained nurse simply to make our burdens lighter or our anxiety less. Show them that as much as ever we look upon our patients as sick and afflicted fellow creatures who need relief, and that we can afford them the help they want in this way better than in any other, but no known means will be omitted or forgotten on our part.

Philips Brooks in his last days said that a minister who was up-to-date was in rather poor business. But give us nurses who are up-to-date. In the care they give the sick and injured, they help us to save the father to his family, the wife and mother to her home, the child to its parents. Some people claim the world is not growing better as it grows older. They say things were not so in their day, and they wish to take us back to the days when the red cradle rocked, and they would ignore the more comfortable, the more sanitary, and less cumbersome methods used in the care of the infant today. The true nurse says not so, let us not do as our forefathers did but let us do our work in a higher, nobler and better way, for the relief of suffering humanity. Is there a class that do a better work? The sun does not shine for itself alone; it is for the race. Nurses do not work for themselves alone; but also for others. The good they do is not all confined to the sick room or to the interior of walls where their work is mostly done. When a nurse graduates some might say her work is done. No more so than the work of the steam engine just completed. It is the number of miles it can make, the number of coaches and passengers it can draw, as it jars the earth with its iron tread during the years which are to come that count. The nurses' sense of duty, like that of the physician is a constant reminder to make them do their best. The once anxious doubt as to the fate or result no longer disturbs the physician's mind and the importance of this cannot be exaggerated, for our only hope in many cases rests in the care which is given the case. The day of memory and thanks at the hands of the medical profession toward the nurses as a class, is not somewhere in the future. It has come now and so we draw a scarlet line below the name of those who have done and are doing so much to help the sick and injured to get well, a line of scarlet below the names of those who have shared our anxiety until the sufferer was relieved. It is safe to assume that in the future, history will point out that many of the brilliant results obtained in both medicine and surgery will be closely associated with the help given by the nursing profession, their help rendering it possible to point out lines of safety and sources of danger. The people that others see strong and well, full of joy and activity, come under our observation and also that of the nurse, when they are sick in both body and mind. Men in other callings and avocations of life see people only when they are well and ready to have their photographs taken, when they are at their best and when the most generous. But the nurse, like the physician, sees people under every disadvantage of disease, sees the strong when they are weak, the beautiful when they are repulsive with suffering which can be relieved only with care. In our success in modern days with the sick and injured who are entrusted to our care, do not let us be exalted more than facts will warrant. Let us give credit and praise to those who in the morning, at noonday, at evening time, were anxiously waiting and watching by the bedside where we had great interests at stake.

